# Biofortified β-carotene rice improves vitamin A intake and reduces the prevalence of inadequacy among women and young children in a simulated analysis in Bangladesh, Indonesia, and the Philippines^1^

**DOI:** 10.3945/ajcn.115.129270

**Published:** 2016-08-10

**Authors:** Fabiana F De Moura, Mourad Moursi, Moira Donahue Angel, Imelda Angeles-Agdeppa, Atmarita Atmarita, Glen M Gironella, Siti Muslimatun, Alicia Carriquiry

**Affiliations:** 2 HarvestPlus, International Food Policy Research Institute, Washington, DC;; 3 Department of Science and Technology, Food and Nutrition Research Institute, Taguig, Philippines;; 4 National Institute of Health Research and Development, Ministry of Health, Jakarta, Indonesia;; 5 Department of Food Science, Indonesia International Institute for Life Sciences, Jakarta, Indonesia; and; 6 Department of Statistics, Iowa State University, Ames, IA

**Keywords:** rice, biofortification, β-carotene, vitamin A, simulation, dietary intake, Bangladesh, Indonesia, Philippines, Asia

## Abstract

**Background:** Vitamin A deficiency continues to be a major public health problem affecting developing countries where people eat mostly rice as a staple food. In Asia, rice provides up to 80% of the total daily energy intake.

**Objective:** We used existing data sets from Bangladesh, Indonesia, and the Philippines, where dietary intakes have been quantified at the individual level to *1*) determine the rice and vitamin A intake in nonpregnant, nonlactating women of reproductive age and in nonbreastfed children 1–3 y old and *2*) simulate the amount of change that could be achieved in the prevalence of inadequate intake of vitamin A if rice biofortified with β-carotene were consumed instead of the rice consumed at present.

**Design:** We considered a range of 4–20 parts per million (ppm) of β-carotene content and 10–70% substitution levels for the biofortified rice. Software was used to estimate usual rice and vitamin A intake for the simulation analyses.

**Results:** In an analysis by country, the substitution of biofortified rice for white rice in the optimistic scenario (20 ppm and 70% substitution) decreased the prevalence of vitamin A inadequacy from baseline 78% in women and 71% in children in Bangladesh. In Indonesia and the Philippines, the prevalence of inadequacy fell by 55–60% in women and dropped by nearly 30% in children from baseline.

**Conclusions:** The results of the simulation analysis were striking in that even low substitution levels and modest increases in the β-carotene of rice produced a meaningful decrease in the prevalence of inadequate intake of vitamin A. Increasing the substitution levels had a greater impact than increasing the β-carotene content by >12 ppm.

## INTRODUCTION

Globally, child deaths annually attributable to vitamin A deficiency are estimated to be 157,000 among preschool children 6–59 mo of age (1). Although the prevalence of clinical symptoms of vitamin A deficiency (e.g., signs of xerophthalmia) has declined, subclinical vitamin A deficiency affects high proportions of children in Africa and Southeast Asia (1). Biofortification, the process of increasing nutrients in food crops, provides a sustainable and long-term strategy for delivering micronutrients (including vitamin A through its precursor β-carotene) to populations in developing countries (2). Conventional breeding methods and the transgenic approach are currently being used to increase the concentrations of micronutrients in food crops (2). Substantial progress has been made in our understanding of whether biofortification is an effective strategy to reduce population micronutrient deficiency by establishing proof of concept for conventionally bred β-carotene–rich orange sweet potato and showing effects on maternal and child intake of vitamin A (3, 4) and child vitamin A status (5).

Rice, which is a staple food crop in many Asian countries, provides up to 80% of the energy intake of the poor; thus, it is a perfect candidate for addressing vitamin A deficiency through biofortification. Germplasm screening did not reveal any cultivar of rice capable of accumulating provitamin A carotenoids in the grain (6). Therefore, increasing provitamin A content could be achieved only through a transgenic approach. In β-carotene rice, commonly known as golden rice because of its yellow hue, 2 genes naturally involved in carotene biosynthesis (7) were inserted into the rice genome by using transgenics. This insertion restarts the carotenoid biosynthetic pathway that is normally inactive, leading to the production of β-carotene in the grain. The current amount of β-carotene in biofortified β-carotene rice is 35 parts per million (ppm),^7^ with an estimated bioconversion rate of 3.8:1 from β-carotene to vitamin A (8). A review on the bioconversion of β-carotene to vitamin A supports the inclusion of dietary interventions with plant sources of β-carotene as a strategy for increasing vitamin A status in populations at risk of deficiency (9). Biofortified β-carotene rice has been backcrossed into popular rice varieties for the Philippines, Indonesia, India, and Bangladesh (6).

The purpose of this study was to understand the potential impact of consumption of biofortified β-carotene rice on the prevalence of inadequate vitamin A intake in populations in which rice is a staple crop. Using national dietary intake data sets from Indonesia (10) and the Philippines (11) and a nutrition survey from 2 districts in Bangladesh (12), we aimed to *1*) determine rice and vitamin A intake in nonbreastfed children 1–3 y of age and in nonpregnant, nonlactating women of reproductive age; and *2*) predict (via simulation) the change in prevalence of inadequate intake of vitamin A when biofortified β-carotene rice is substituted for white rice in their typical diet, applying a range of β-carotene content of 4–20 ppm and substitution levels of 10–70% of biofortified rice.

## METHODS

We included women of reproductive age (14–50 y) and nonbreastfed children aged 1–3 y. We did not have measures of breast milk consumption in our dietary data; thus, breastfeeding children were excluded from this analysis. In this article, we present results from nonpregnant, nonlactating women. We used dietary data from 3 surveys: the 7th National Nutrition Survey in the Philippines (11), the Indonesian Food Consumption Survey 2010 (10), and a nutrition survey conducted in 2 districts in Bangladesh (12). The survey methods are briefly described.

### Indonesia data set

The Indonesian Food Consumption Survey 2010 (10) is part of the Basic Health Research study, referred to as RISKESDAS 2010. It is a nationally representative cross-sectional study with the aim of measuring achievements of the Millennium Development Goals. With the use of a 2-stage sampling design, 823 of 2800 census blocks were randomly selected for food consumption data collection. A total of 20,575 households participated. Food consumption was measured with a single 24-h recall for each individual within a household. Before data collection, enumerators purchased foods in the local market and converted household measures of foods into weights in grams.

### Philippines data set

The 7th National Nutrition Survey data from 2008 is the 7th iteration of a nationally representative survey conducted every 5 y to assess the health and nutritional status of the Filipino population (11). A multistage sampling design was used, and 4880 households were selected to complete the dietary component of the survey. Food intake was estimated by using the 24-h recall method collected on 2 nonconsecutive days. Foods consumed were estimated with household measures and converted into weights in grams.

### Bangladesh data set

A dietary intake survey was performed in 2 rural districts in northern Bangladesh, Trishal (October 2007 to May 2008) and Pirgaccha (January–June 2008), to assess the potential of a biofortified rice program (12). The selection of the districts was based on high rice consumption and high prevalence of poverty and food insecurity. A multistage, cluster sampling design was performed to select a representative sample of 240 mother and child pairs from each district from which a total of 237 women were nonpregnant and nonlactating and 77 children were 1–3 y old and nonbreastfeeding. Dietary intakes were assessed by direct observation in the homes by using 12-h weighed food records and a recall of additional foods consumed during the subsequent 12-h period. Two nonconsecutive days of dietary information were obtained over 1 wk.

### Parameters for simulation

We assumed the β-carotene to vitamin A bioconversion of 3.8:1 for biofortified β-carotene rice (8) and 12:1 for all remaining foods. Fortified foods were considered in this analysis as part of the baseline vitamin A intake. This is especially true for the Philippines, where fortified foods were encountered more frequently than in Bangladesh or Indonesia. For cooked rice grains, we factored in a 5% loss of vitamin A due to cooking and 10% for rice flour or products based on rice flour (M Moursi, unpublished results, 2013). We applied the following estimated average requirements (EAR) to determine prevalence of inadequate vitamin A intakes for each group: for nonpregnant, nonlactating women, 485 μg/d (14–18 y old) and 500 μg/d (19–50 y old); for children 1–3 y old, 210 μg/d (13).

For a given level of biofortification, adopters were selected at random from each population subgroup in this simulation analysis. For example, to simulate 10% adoption of biofortified rice, we randomly selected 10% of individuals in a group and substituted their entire rice intake with biofortified rice. We repeated this process 10 times for each β-carotene and adoption level. Usual rice, energy, and vitamin A intake and the prevalence of vitamin A inadequacy were then estimated and averaged over the 10 independent simulation replicates. By randomly selecting a different subset of adopters in each replication, we are able to average the effect of biofortification and adoption levels across the different subsamples.

### Statistical analysis

We estimated usual intake of rice, energy, and vitamin A using an approach developed at Iowa State University (14). This method requires that observed intakes be collected over a minimum of 2 nonconsecutive days for at least a subsample of participants to permit estimation of the within-person variance component in intake. Because only one 24-h recall was collected in the Indonesia survey, we applied the same ratio of the within-to-the-between variance in vitamin A intake found in the Philippines data set to estimate the usual intake distributions of women and children in Indonesia. We estimated the prevalence of inadequate vitamin A intake using the EAR cutoff method (15, 16), which states that the prevalence of inadequate intake can be estimated as the proportion of individuals with usual intakes below the EAR. Females aged 14–50 y were separated into 3 age groups for analysis: 14–18 y, 19–30 y, and 31–50 y. This was done because of different EAR requirements as well as for computing efficiency of PC-Side (Iowa State University). We combined the results obtained from the 3 groups of women by computing weighted averages of results in which the weights are sample frequencies of each subgroup. To explore the effect of different assumptions about the bioconversion factors for β-carotene on the effect of biofortifying rice, we conducted a sensitivity analysis comparing the bioconversion factor 3.8:1 (optimistic assumption) used in our analysis with an intermediate assumption of a 6:1 conversion ratio (17) and a pessimistic assumption of a 12:1 conversion ratio (13) for nonpregnant, nonlactating women and children in the Philippines.

We used software developed by the Department of Statistics and Statistical Laboratory at Iowa State University called PC-Side to implement the methodology (14). All figures were constructed by using R version 3.1.3 (The R Foundation for Statistical Computing).

## RESULTS

Our simulation study included a total sample size of 65,678 women and 6945 children from Indonesia, 4242 females and 939 children from the Philippines, and 237 women and 77 children from Bangladesh (**Table 1**).

**TABLE 1:** Baseline estimated usual energy, rice, and vitamin A intakes and prevalence of inadequate vitamin A intake in women and young children in Bangladesh, Indonesia, and the Philippines^1^

		Usual energy intake,^2^ kcal/d	Usual rice intake,^2^ g/d		Usual vitamin A intake,^2^^,^^3^ μg RAE/d		
	*N*	Median (25th, 75th percentiles)	Mean ± SE [% rice consumers]	Median (25th, 75th percentiles)	Mean ± SE	Median (25th, 75th percentiles)	Prevalence of inadequate vitamin A intake,^4^ %
Bangladesh							
Women	237	1796 (1485, 2198)	419 ± 8.4 [100]	417 (361, 475)	219 ± 15.1	179 (110, 287)	93
Children	77	932 (745, 1113)	148 ± 5.8 [100]	146 (112, 182)	100 ± 8.1	83 (51, 130)	93
Indonesia							
Women	65,678	1147 (882, 1480)	163 ± 0.4 [98]	160 (125, 198)	426 ± 1.8	387 (261, 551)	68
Children	6945	817 (578, 1107)	85 ± 0.02 [96]	72 (44, 108)	307 ± 2.1	281 (175, 411)	34
Philippines							
Women	4242	1519 (1130, 1987)	280 ± 0.12 [100]	269 (196, 355)	380 ± 8.3	300 (197, 506)	74
Children	939	666 (465, 943)	78 ± 0.06 [100]	109 (62, 175)	335 ± 10.4	250 (123, 446)	43

1 Women were aged 14–50 y; children were aged 1–3 y. EAR, estimated average requirement, RAE, retinol activity equivalent.

2 Usual energy, rice, and vitamin A intakes were estimated by using the Iowa State University method (16) with the PC-Side program.

3 Vitamin A intakes were calculated by using RAEs.

4 Prevalence of inadequacy was based on the EAR of 485 μg vitamin A/d for nonpregnant, nonlactating females (14–18 y old) and 500 μg vitamin A/d for women (19–50 y old), and 210 μg vitamin A/d for children 1–3 y old (13).

Table 1 presents usual energy, rice, and vitamin A intake in women and children and prevalence of vitamin A inadequacy. Usual energy intake [median (25th, 75th percentiles)] in Bangladesh was within expected ranges among women [1796 kcal (1485, 2198 kcal)] and children [932 kcal (745, 1113 kcal)] but unusually low among women in Indonesia [1147 kcal (882, 1480 kcal)]. Usual rice consumption (mean ± SE raw, uncooked weight) among women was highest in Bangladesh (419 ± 8.4 g/d), followed by the Philippines (280 ± 0.12 g/d), and Indonesia (163 ± 0.4 g/d); among children, usual rice consumption was highest in Bangladesh (148 ± 5.8 g/d), followed by Indonesia (85 ± 0.02 g/d), and the Philippines (78 ± 0.06 g/d). Mean ± SE usual vitamin A intake among women and children was lowest in Bangladesh, 219 ± 15.1 and 100 ± 8.1 μg retinol activity equivalents (RAEs)/d, respectively, followed by the Philippines, 380 ± 8.3 and 335 ± 10.4 μg RAE/d, respectively, and Indonesia, 426 ± 1.8 and 307 ± 2.1 μg RAE/d, respectively. Overall, the prevalence of inadequate vitamin A intake at baseline was considered moderate to high depending on the country and target population. Bangladesh presented the highest prevalence of inadequate vitamin A intake (93%) for both women and children. In the Philippines and Indonesia, the prevalence of inadequate vitamin A intake was higher among women (74% and 68%, respectively) than among children (43% and 34%, respectively).

**FIGURE 1:**
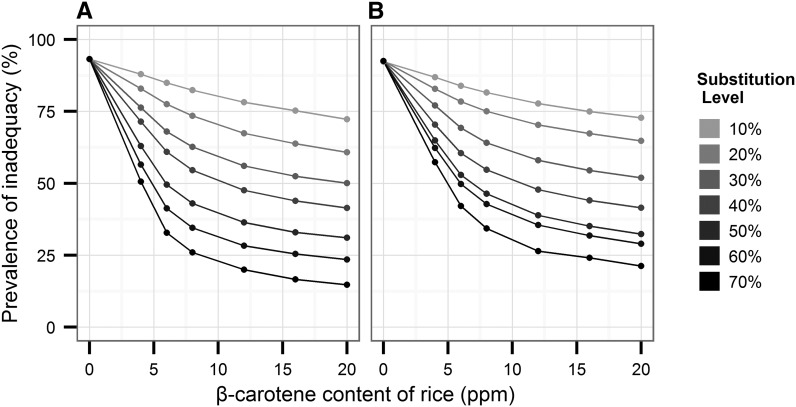
Simulations of the potential impact of biofortified β-carotene rice on the prevalence of inadequate vitamin A intake in Bangladeshi women and young children from 2 rural districts. Various scenarios are depicted in the graphics, showing population adoption rates ranging from 10% to 70% of biofortified rice and ppm levels of β-carotene ranging from 0 to 20 ppm. Simulations were conducted for nonpregnant, nonlactating women of reproductive age (14–50 y old) (A) and nonbreastfeeding children 1–3 y old (B). The Institute of Medicine’s estimated average requirements of vitamin A for each age group were used to calculate probability of inadequate intakes. ppm, parts per million.

**FIGURE 2:**
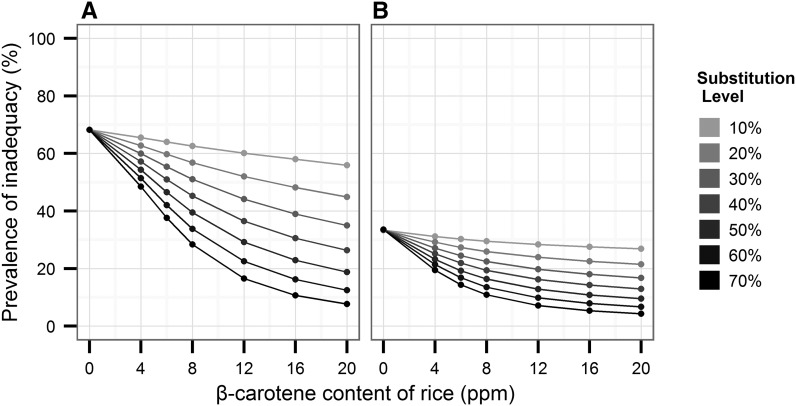
Simulations of the potential impact of biofortified β-carotene rice on the prevalence of inadequate vitamin A intake among Indonesian women and young children. Various scenarios are depicted in the graphics, showing population adoption rates ranging from 10% to 70% of biofortified rice and ppm levels of β-carotene ranging from 0 to 20 ppm. Simulations were conducted for nonpregnant, nonlactating women of reproductive age (14–50 y old) (A) and nonbreastfeeding children 1–3 y old (B). The Institute of Medicine’s estimated average requirements of vitamin A for each age group were used to calculate probability of inadequate intakes. ppm, parts per million.

**FIGURE 3:**
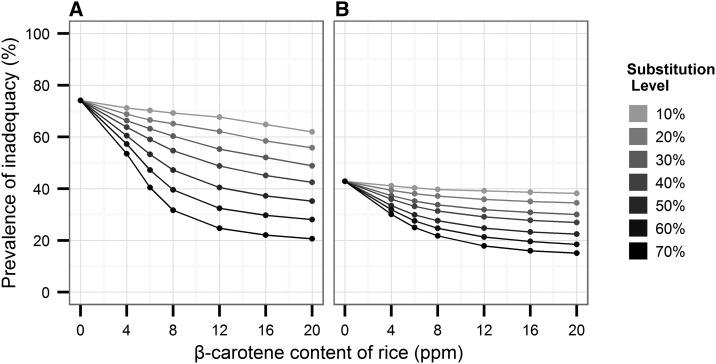
Simulations of the potential impact of biofortified β-carotene rice on the prevalence of inadequate vitamin A intake among Filipino women and children. Various scenarios are depicted in the graphics, showing population adoption rates ranging from 10% to 70% of biofortified rice and ppm levels of β-carotene ranging from 0 to 20 ppm. Simulations were conducted for nonpregnant, nonlactating females of reproductive age (14–50 y old) (A) and nonbreastfeeding children 1–3 y old (B). The Institute of Medicine’s estimated average requirements of vitamin A for each age group were used to calculate probability of inadequate intakes. ppm, parts per million.

**Figures 1**–**3** show predicted reductions in the prevalence of inadequate vitamin A intake for Bangladesh, Indonesia, and the Philippines, with increasing proportions of the population substituting biofortified β-carotene rice for white rice and at various concentrations of β-carotene. In an analysis by country, the substitution of biofortified rice for white rice in the optimistic scenario (20 ppm and 70% substitution) decreased the prevalence of vitamin A inadequacy from baseline 78% in women and 71% among children in Bangladesh (Figure 1). Prevalence of inadequacy fell 55–60% from baseline among Indonesian women (Figure 2A) and Filipino females (Figure 3A). The prevalence of inadequacy in children dropped nearly 30% from baseline in the most optimistic scenario in both Indonesian (Figure 2B) and Filipino children (Figure 3B), driving down the prevalence of inadequacy to <5% in Indonesian children and to 15% in Filipino children.

In an analysis of the impact of the β-carotene content of biofortified rice compared with the substitution level on the reduction of the prevalence of inadequate vitamin A intake, a ≤4-ppm β-carotene content had a small impact on the prevalence of inadequacy at the 10% substitution level and larger impact as substitution levels increased from 10% to 70%. In Indonesia and the Philippines, impacts from 4 ppm biofortified rice ranged from 2% (10% substitution rate) to 23% (70% substitution rate) reduction from baseline prevalence. In Bangladesh, percentage point reductions in the prevalence of inadequate vitamin A intake were estimated at almost 42% among women and 35% among children at the 70% substitution level. Between 12 and 20 ppm, the reductions in inadequate intakes were minimal because of increasing β-carotene concentration alone, with substantial reductions in population prevalence of inadequacy due primarily to increasing substitution levels.

**FIGURE 4:**
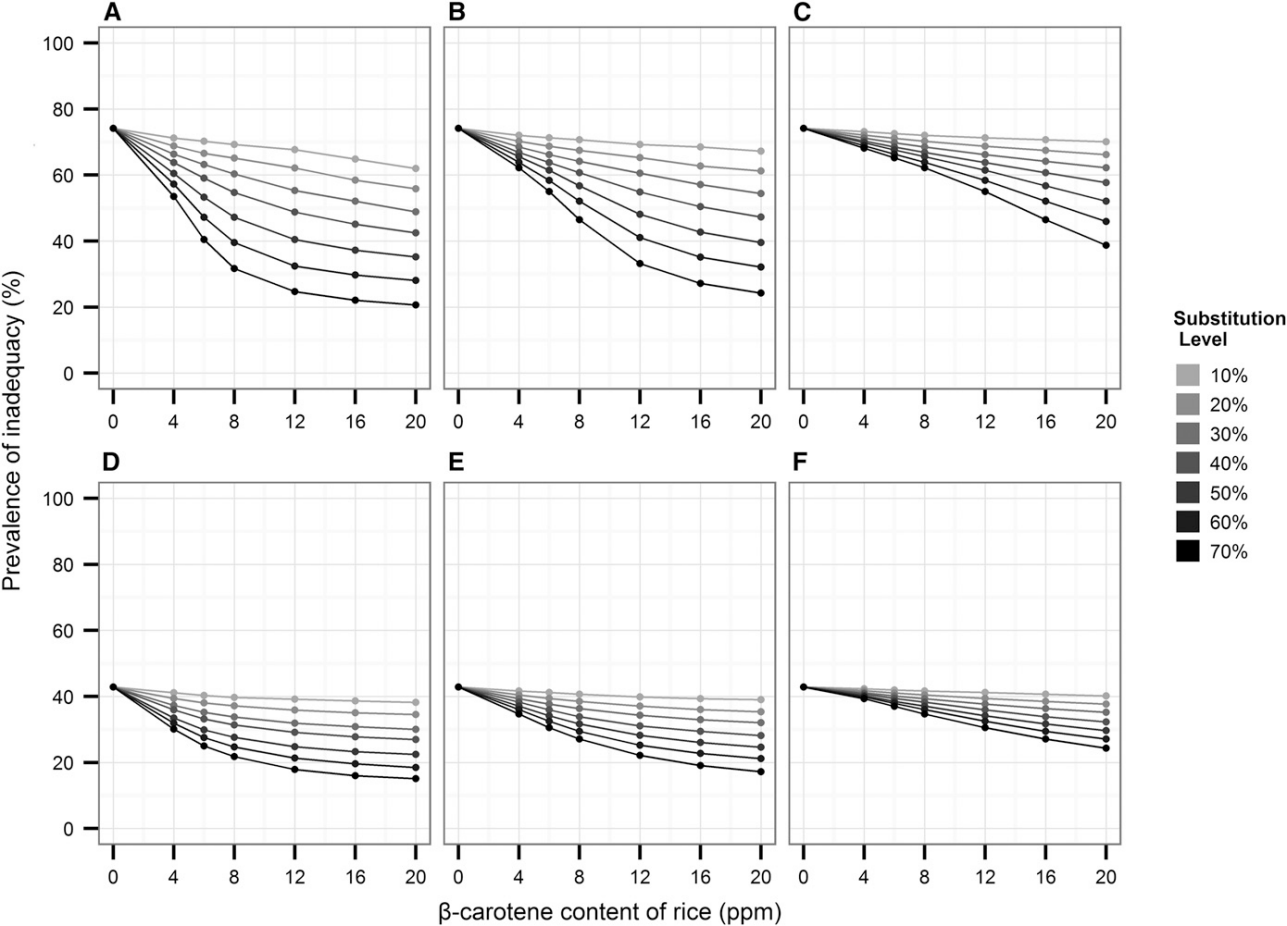
Sensitivity analysis showing how the bioconversion assumption of β-carotene to vitamin A in biofortified rice influences the predicted impact on prevalence of inadequate vitamin A intake in Filipino children (1–3 y old) and nonpregnant and nonlactating women (14–50 y old). For women, panel A shows the original optimistic assumption of a 3.8:1 conversion ratio; panel B shows the intermediate assumption of a 6:1 conversion ratio; and panel C shows a pessimistic assumption of a 12:1 conversion ratio. For children, panels D–F show the optimistic 3.8:1 conversion ratio, the intermediate assumption of 6:1 conversion ratio, and the pessimistic assumption of 12:1 conversion ratio, respectively. ppm, parts per million.

The results from the sensitivity analysis revealed the magnitude of the impact by assuming different bioconversion factors of β-carotene to vitamin A in biofortified rice, from the most optimistic scenario (3.8:1) used in our simulation analysis to the intermediate (6:1) and pessimistic scenarios (12:1) among women (**Figure 4**A–C) and children (Figure 4D–F) from the Philippines. In our analysis, we observed that the overall impact of an intervention among Filipino children was lower if we assumed that the biofortified rice had a 12:1 bioconversion rate, as seen in the smaller spread of the curves (Figure 4D–F). However, there was a small difference in predicted impact when assuming a 6:1 bioconversion rate compared with the 3.8:1 bioconversion factor. Starting at 30% substitution level, different ppm levels resulted in 2% and 5% smaller reductions in the prevalence of inadequacy with 6:1 and 12:1 bioconversion factors, respectively, compared with the original bioconversion factor (3.8:1). At the 50–70% substitution levels, the 6:1 bioconversion factor similarly resulted in 2–5% smaller reductions, whereas the 12:1 bioconversion assumptions resulted in 9–12% smaller reductions in the prevalence of inadequacy than with the original bioconversion rate. For example, in the most optimistic scenario tested (20 ppm and 70% substitution level) and assuming a 12:1 bioconversion factor, the prevalence of inadequacy among children is 24.3%. The expected impact would be much greater with the 6:1 or 3.8:1 bioconversion factors, dropping the prevalence of inadequacy to 17.2% or 15.9%, respectively. We observed similar results for Filipino women (Figure 4A–C).

## DISCUSSION

The greatest simulated impact of a biofortified rice intervention on the prevalence of inadequate vitamin A intake was among Bangladeshi women and among children, with the prevalence of inadequate intake dropping from 93% at baseline to ∼20% and 13% among children and women, respectively. In Indonesia, the impact was less pronounced, particularly among children, for whom the baseline prevalence of inadequate intake was not as high (34%). Even in this case, however, the most optimistic scenario of 20 ppm β-carotene at the 70% substitution level resulted in <10% of the population with inadequate vitamin A intakes. For Filipino women, although baseline prevalence of inadequate intake was similar to that of Indonesian women, the most optimistic scenario reduced the population prevalence of inadequate intake to 25%. Despite these differences, which can largely be explained by differences in rice consumption per capita and per day, in most cases the greater substitution levels had a much greater impact on driving the reduction of the prevalence of inadequate intakes when compared with increasing β-carotene levels to >12 ppm, as noted by the flattening of slopes. This suggests that, to maximize their impact, programs will have to put more emphasis on encouraging the adoption of biofortified β-carotene rice among farmers and subsequently creating a demand for it on the consumer side to drive both adoption and consumption.

In our study, we used a conservative range of β-carotene content, 4–20 ppm, which takes into account the degradation of β-carotene during the storage of rice. Although the most recent lines of β-carotene rice contain up to 35 ppm of β-carotene (7), there is a steep degradation (up to 50%) of β-carotene during the first 2 wk when rice is stored at room temperature, which then stabilizes, as was also observed with biofortified provitamin A maize (12). In this simulation analysis, we also used the EAR cutoff method to determine the prevalence of inadequate intakes of vitamin A for populations. The cutoff method is widely used in food fortification programs, and a detailed explanation of the method is provided in the WHO Guidelines on Food Fortification with Micronutrients (17). Of importance, the goal of food-based approaches to reducing micronutrient deficiency is to shift the nutrient intake distribution such that the prevalence of inadequate intake is minimized without putting the population at risk of excess intake. In our most optimistic scenario of 20 ppm and a 70% substitution level, usual vitamin A intake was as high as 838 μg/d, which is lower than the upper-limit intake of 3,000 μg/d of vitamin A established by the Institute of Medicine when consumed as preformed vitamin A (13). Furthermore, the biofortified rice contains β-carotene, for which an upper limit has not been established (18), because β-carotene does not present the toxic effects observed with excessive intake of preformed vitamin A. In the case of an individual with a high vitamin A liver store, the body will downregulate the cleavage of β-carotene to vitamin A, absorbing it as intact β-carotene (19).

Our study has some strengths and weaknesses. Among the strengths is the 7-point substitution level used in our simulation analysis, where previous simulation studies did not consider a wide range of substitution levels. In previous studies, usually 100% of the biofortified crop is substituted for the nonbiofortified crop in the analysis (20), and in some instances 2 (21) or 3 (12) substitution levels were applied, at most. We were also conservative in the β-carotene content assumed, reflecting the degradation that occurs during storage. And finally, our sensitivity analysis demonstrated that the 6:1 bioconversion factor had a small impact (<5%) on the reduction of the prevalence of inadequate intake of vitamin A (Figure 4) when compared with the 3.8:1 bioconversion factor. Although the 12:1 bioconversion factor had a higher impact, 10–12%, a review on bioavailability of biofortified provitamin A crops (22) has shown that the bioconversion of biofortified cereal crops ranged between 3:1 and 7:1, which is closer to the 6:1 bioconversion than the average 12:1, derived mainly from vegetables (13).

Most of our study’s weaknesses are related to the dietary data sets and are inherent to the current tools used for dietary assessment. Representation was the main concern for Bangladesh, with the dietary data representing only 2 districts in the northern region of the country known for high consumption of rice and poor nutrition status. Results of rice consumption for women were found to be very similar to the per capita consumption estimates (453 g/d) from the 2010 Household Income and Expenditure Survey (HIES) of 12,240 rural households in Bangladesh (23). However, the vitamin A–inadequate intake in the Bangladesh study (93%) was much higher than the reported 61% in the 2010 HIES. This may be explained in part by the fact that the 2010 HIES collected data only at the household level and apportioned intakes to household members by using estimated energy requirements based on sex and age. Even if this approach permits approximating energy intake at the individual level, it is not necessarily the case that micronutrient intake is well apportioned. Another explanation may be that vitamin A intake changes substantially depending on the season and availability of foods rich in vitamin A or carotenoids in different regions, whereas rice intake is more evenly distributed across regions and seasons. Therefore, the results of our simulation would be most relevant to those areas in the country where the inadequacy of vitamin A is high.

In the case of the Philippines and Indonesia, national surveys were used; however, rice consumption was low, and we suspect that underreporting might have occurred as indicated by the low reported usual energy intake of 1519 and 1147 kcal/d (Table 1) among women in the Philippines and Indonesia, respectively. Day-to-day variation, which is the main source of random error of a dietary recall, could not be accounted for in the Indonesian survey because only one 24-h recall was collected. Although intakes of rice among Indonesian and Filipino women and children are likely underreported, our simulations show that substantial decreases in the prevalence of inadequacy can be achieved with relatively low rice consumption. Interestingly, the lowest estimation of prevalence of inadequate vitamin A intake that could be achieved in our most optimistic scenario (20 ppm of β-carotene rice at a 70% substitution level) was among Indonesian women and children, dropping to <10% (Figure 2). This is attributable, in part, to the shape of the population distribution of vitamin A intakes, as well as relatively low baseline prevalence of vitamin A inadequacy. Because all our biofortification scenarios compare baseline and postbiofortification intakes, our general conclusions regarding impact are unaffected, except for the fact that the baseline prevalence of inadequacy may be overestimated. As described in the methods section, adopters were selected at random from among survey participants in each age group, but it is likely that household and individual characteristics are associated with the propensity to adopt a transgenic crop. Therefore, the adoption model can be improved if information about the factors that affect adoption of biofortified crops becomes available.

In summary, biofortified β-carotene rice can substantially increase vitamin A intake and consequently reduce the prevalence of inadequacy of this vitamin. Increasing vitamin A intake through biofortified rice at 8–12 ppm of β-carotene, in combination with programs that increase adoption of biofortified rice in a population, can be an effective method at reducing population prevalence of inadequate vitamin A intakes. Our results showed, however, that increasing the β-carotene beyond 12 ppm has little added benefit; rather, public health programs will have the most impact by increasing the substitution of white rice by biofortified β-carotene rice.

## Footnotes

7 Abbreviations used: EAR, estimated average requirement; HIES, Household Income and Expenditure Survey; ppm, parts per million; RAE, retinol activity equivalent.
